# Plasma soluble vascular endothelial growth factor receptor-1 levels predict outcomes of pneumonia-related septic shock patients: a prospective observational study

**DOI:** 10.1186/cc9412

**Published:** 2011-01-10

**Authors:** Kuang-Yao Yang, Kuan-Ting Liu, Yu-Chun Chen, Chun-Sheng Chen, Yu-Chin Lee, Reury-Perng Perng, Jia-Yih Feng

**Affiliations:** 1 Chest Department, Taipei Veterans General Hospital, Shipai Road, Taipei 112, Taiwan, ROC; 2 Institute of Clinical Medicine, School of Medicine, National Yang-Ming University, Linong Street, Taipei 112, Taiwan, ROC; 3 Taipei City Hospital, Zhongxiao Branch, Tung Teh Road, Taipei 115, Taiwan, ROC; 4 School of Medicine, National Yang-Ming University, Linong Street, Taipei 112, Taiwan, ROC; 5 Institute of Medical Biometry and Informatics, University of Heidelberg, Im Neuenheimer Feld 305, Heidelberg 69120, Germany

## Abstract

**Introduction:**

Despite recent advances in the management of septic shock, mortality rates are still unacceptably high. Early identification of the high-mortality risk group for early intervention remains an issue under exploration. Vascular endothelial growth factor (VEGF), soluble vascular endothelial growth factor receptor-1 (sVEGFR1) and urokinase plasminogen activator (uPA) have diverse effects in the pathogenesis of sepsis, which involve pro-inflammation, anti-inflammation, endothelial cell repair, and vascular permeability change. Their roles in predicting mortality and organ dysfunction remain to be clarified.

**Methods:**

Pneumonia-related septic shock patients from medical intensive care units were enrolled for this prospective observational study. We also included 20 patients with pneumonia without organ dysfunction for comparison. Plasma levels of VEGF and sVEGFR1 and uPA activity within 24 hours of shock onset were measured. We compared plasma levels of these biomarkers with APACHE II scores between subgroups of patients, and evaluated their predictive value for 28-day mortality and organ dysfunction.

**Results:**

A total of 101 patients, including 81 with pneumonia-related septic shock and 20 with pneumonia without organ dysfunction, were enrolled. Non-survivors of septic shock had significantly higher plasma sVEGFR1 levels (659.3 ± 1022.8 vs. 221.1 ± 268.9 pg/mL, respectively, *P *< 0.001) and uPA activity (47.2 ± 40.6 vs. 27.6 ± 17.2 units, respectively, *P *= 0.001) when compared with those of the survivors. Kaplan-Meier survival analysis demonstrated significantly higher mortality in patients with higher levels of sVEGFR1 (P < 0.001) and uPA activity (P = 0.031). In Cox regression analysis, plasma sVEGFR1 level was independently associated with, and best predicted, the 28-day mortality of septic shock (HR: 1.55, 95% CI: 1.05-2.30). Plasma sVEGFR1 level and uPA activity had good correlation with renal dysfunction, metabolic acidosis, and hematologic dysfunction; their levels significantly increased when the number of organ dysfunctions increased. In multivariate analysis, plasma sVEGFR1 level (HR: 2.82, 95% CI: 1.17-6.81) and uPA activity (HR: 2.75, 95% CI: 1.06-7.13) were independent predictors of the presence of concomitant multi-organ dysfunction. The predictive value of VEGF for mortality and organ dysfunction was limited in pneumonia-related septic shock patients.

**Conclusions:**

High plasma sVEGFR1 level in the early stage of pneumonia-related septic shock independently predicted 28-day mortality and multi-organ dysfunction.

## Introduction

Sepsis occurs as a result of a complex interaction between the microorganism and the host immune response, and systemic inflammatory response syndrome is an important feature of sepsis [1,2]. Septic shock, defined as sepsis combined with hypotension that is refractory to fluid resuscitation, is the main cause of death in patients with sepsis [3]. Even with advances in current management, the mortality rate of septic shock has remained around 40% to 70% [4,5]. Determination of novel markers that are present in the early phase of septic shock and that have good correlations with outcome is essential for the management of septic shock. These markers would not only help identify patients with an extraordinarily high mortality risk (and who thus deserve aggressive management) but also provide potential therapeutic targets.

Endothelial cell dysfunction and disturbance of the coagulation system have been proposed to be pivotal factors in the pathophysiology of sepsis [2,6]. Vascular endothelial growth factor (VEGF) is a glycoprotein that is synthesized and released by vascular endothelial cells, lung epithelium, platelets, and leukocytes [7]. Through binding with the VEGF receptor, VEGF can enhance angiogenesis and increase microvascular permeability, which may lead to edema and hypotension [8,9]. VEGF also has been found to promote the proliferation, migration, and survival of endothelial cells [10]. Three types of VEGF receptors - fms-like tyrosine kinase (FLT-1, VEGFR1), kinase-insert-domain-containing receptor (KDR, VEGFR2), and fms-like tyrosine kinase-4 (Flt-4, VEGFR3) - have been reported and are expressed mostly on endothelial cells [11,12]. Soluble VEGFR1 (sVEGFR1) is generated by alternative splicing of VEGFR1 mRNA and functions as an intrinsic negative counterpart of VEGF signaling [13]. Recently, animal and human studies have reported controversial results regarding the association between VEGF, sVEGFR1 concentration, and disease severity in sepsis and septic shock [14-17].

Urokinase plasminogen activator (uPA) is a serine protease that catalyzes the conversion of plasminogen to plasmin. In addition to having a role in fibrinolysis, uPA has been described as being involved in the inflammatory process and endothelial cell migration [18-20]. Furthermore, recent studies have indicated that uPA plays a vital role in the process of VEGF-induced vascular permeability change [21], which may involve the mechanism of septic shock. Despite the complex role of uPA in sepsis, the impact of uPA on the outcome of septic shock remains to be identified.

The purpose of this study was to evaluate the role of endothelial cell-related biomarkers, including VEGF, sVEGFR1, and uPA, with respect to the mortality of patients with pneumonia-related septic shock. The predictive values of these markers for disease severity and organ dysfunction were also investigated.

## Materials and methods

### Patients

This prospective, observational study was conducted at Taipei Veterans General Hospital, a tertiary medical center in Taiwan. From January 2006 to February 2008, all patients who were at least 18 years of age and who were admitted to the medical intensive care unit (ICU) and respiratory ICU were screened. Patients who had a diagnosis of pneumonia and who fulfilled the American College of Chest Physicians/Society of Critical Care Medicine criteria for septic shock [1], which is defined as refractory hypotension despite adequate fluid supplement and the requirement of vasopressors to maintain the mean arterial blood pressure of at least 65 mm Hg, were eligible for enrollment. The diagnosis of pneumonia was confirmed on the basis of typical clinical presentations, fever, leukocytosis, and new infiltrates on chest radiographs [22,23]. Patients with underlying malignancies, autoimmune disorders, active thromboembolic disease and those who failed to give informed consent were excluded. During the same period, we also enrolled 20 patients who were from the general medical ward and who had a diagnosis of pneumonia without sepsis or organ dysfunction as a control group. All patients were treated in accordance with the current treatment guidelines, and broad-spectrum antibiotics were administered within 1 hour of the onset of septic shock, as is routine in our ICUs. The antibiotics were also adjusted on the basis of the clinical response and the susceptibility profile of bacterial cultures. All patients with septic shock received a physiological dose of corticosteroids (hydrocortisone, 200 mg/day, divided in four doses). The study protocol was approved by the Taipei Veterans General Hospital Institutional Review Board, and the study was conducted in accordance with the Declaration of Helsinki. Written informed consent was obtained from all participants or their authorized representatives before enrollment.

### Clinical evaluation

Demographic characteristics, underlying comorbidities, disease severity, and organ dysfunction were determined on the day of enrollment. Organ dysfunction was defined as in a previous study [24]; the details of the definition are provided as supplemental material (Additional file 1). Disease severity was evaluated by the Acute Physiology and Chronic Health Evaluation II (APACHE II) score on the day of enrollment. Multi-organ dysfunction was defined as septic shock plus one or more organ dysfunctions, and the severity of multi-organ dysfunction was evaluated by the Sequential Organ Failure Assessment (SOFA) score. Survival status at 28 days and beyond was determined.

### Blood sampling and biomarker measurement

Blood samples were collected from peripheral vessels within 24 hours of shock onset and study entry. Plasma was separated from whole blood and stored at -70°C until analysis. Total forms of plasma VEGF and sVEGFR1 concentrations were measured in duplicate with commercial enzyme-linked immunosorbent assay kits (Quantikine; R&D Systems, Inc., Minneapolis, MN, USA). Plasma uPA activity was evaluated with a uPA Activity Assay kit (Chemicon International, Temecula, CA, USA).

### Statistical analysis

Statistical analysis was performed with SPSS 14.0 software (SPSS, Inc., Chicago, IL, USA). Continuous variables between subgroups were compared with the Mann-Whitney *U *test or independent *t *test, and categorical variables were compared using Pearson's chi-square test. Binary logistic regression analysis was performed to determine the independent variables for multi-organ dysfunction. To determine the predictive accuracy of biomarker levels for survival and organ dysfunction, receiver operating characteristic (ROC) curves were constructed, and the areas under the curves (AUCs) were calculated.

For survival analysis, patients were stratified into subgroups according to plasma biomarker levels. Survival time was estimated by the Kaplan-Meier method, and the log-rank test was used to compare mortality between patients with quartile biomarker levels. Censored analysis was used since observation time was limited by discharge from the hospital. A multivariate Cox proportional hazards regression model with forward stepwise selection procedures was used to identify the risk factors for 28-day mortality. A *P *value of less than 0.1 in the univariate analysis was required for a variable to enter the multivariate model. A *P *value of less than 0.05 was considered statistically significant for all tests.

## Results

### Patient characteristics

**Figure 1:**
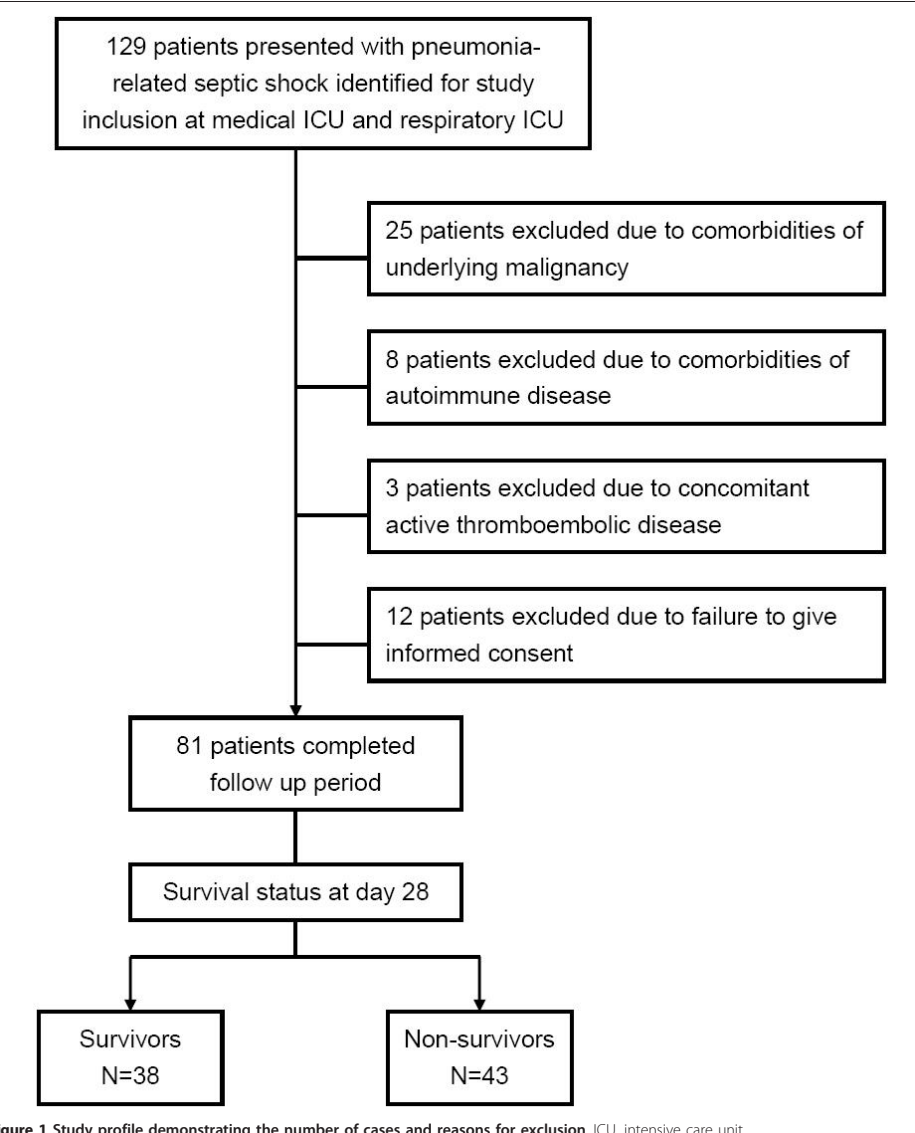
**Study profile demonstrating the number of cases and reasons for exclusion**. ICU, intensive care unit.

The study profile showing the number of cases and reasons for exclusion is shown in Figure 1. Ultimately, 81 patients with pneumonia complicated with septic shock were enrolled in the study. We also enrolled 20 patients with pneumonia without organ failure for comparison; all of these patients survived and were successfully discharged from the hospital. Among the 81 pneumonia patients with septic shock, 43 died within 28 days (28-day mortality of 53.1%), 11 died during their hospital stay after day 28 (hospital mortality of 66.7%), and 27 (33.3%) survived and were discharged from the hospital. The demographic characteristics of all pneumonia patients are shown in Table 1. The mean age of these patients was 79.3 ± 10.9 years, and the majority were male (87/101, 86.1%). There were no differences in age, gender, and underlying comorbidities between the patients with pneumonia without organ dysfunction, the septic shock survivors, and the non-survivors at day 28. The non-survivors of pneumonia-related septic shock had a significantly higher APACHE II score (*P *< 0.001), lower PaO_2_/FiO_2 _(arterial partial pressure of oxygen/fraction of inspired oxygen) ratio (*P *< 0.001), higher SOFA score (*P *< 0.001), and more organ dysfunctions as compared with the survivors. Pathogens were identified more frequently in patients with pneumonia-related septic shock as compared with those without organ dysfunction (*P *< 0.001).

**Table 1:** Demographic characteristics of patients with pneumonia without organ dysfunction and those with pneumonia-related septic shock

		**Pneumonia with septic shock**^**a**^		
		
	Pneumonia without organ dysfunction	Survivors	Non-survivors	***P *value**^**b**^
Number of patients	20	38	43	
Age, years	77.2 (14.1)	79.9 (7.9)	79.7 (11.7)	0.93
Male gender	18 (90%)	32 (84.2%)	37 (86%)	0.81
Comorbidity				
Obstructive airway disease	3 (15%)	10 (26.3%)	9 (20.9%)	0.57
Interstitial lung disease	1 (5%)	6 (15.8%)	5 (11.6%)	0.59
Congestive heart failure	1 (5%)	5 (13.2%)	4 (9.3%)	0.58
Diabetes mellitus	6 (30%)	10 (26.3%)	14 (32.6%)	0.54
Chronic renal insufficiency	1 (5%)	5 (13.2%)	9 (20.9%)	0.36
Pathogens in sputum culture				
Gram-positive bacteria	3 (15%)	10 (26.3%)	8 (18.6%)	0.41
Gram-negative bacteria	3 (15%)	32 (84.2%)	30 (69.8%)	0.12
Pathogens in blood culture				
Gram-positive bacteria	0	4 (10.5%)	1 (2.3%)	0.18
Gram-negative bacteria	0	4 (10.5%)	11 (25.6%)	0.08
Source of pneumonia				0.38
Community-acquired pneumonia	20 (100%)	24 (63.2%)	21 (48.8%)	
Hospital-acquired pneumonia	0	7 (18.4%)	9 (20.9%)	
Ventilator-acquired pneumonia	0	7 (18.4%)	13 (30.2%)	
Disease severity				
APACHE II score	-	24.1 (5.1)	33.4 (7.1)	<0.001
PaO_2_/FiO_2 _ratio	-	214.2 (87.6)	156.8 (100.3)	<0.001
Organ dysfunction^c^				
Renal dysfunction	-	10 (26.3%)	28 (65.1%)	<0.001
Hematologic dysfunction	-	7 (18.4%)	20 (46.5%)	0.007
Metabolic acidosis	-	3 (7.9%)	20 (46.5%)	<0.001
Adult respiratory distress syndrome	-	18 (47.4%)	32 (74.4%)	0.012
Number of organ dysfunctions				
≥2 organ failures (including shock)	-	23 (60.5%)	39 (90.7%)	0.001
≥3 organ failures (including shock)	-	5 (13.2%)	29 (67.4%)	<0.001
≥4 organ failures (including shock)	-	2 (5.3%)	19 (44.2%)	<0.001
SOFA score	-	10.6 (2.5)	12.5 (2.6)	<0.001
Hospital length of stay, days	8.4 (3.6)	64.7 (40.5)	22.4 (15.4)	<0.001
Intensive care unit length of stay, days	-	40.6 (25.1)	18.3 (12.0)	<0.001
Combined with other infection sources	-	8 (21.1%)	6 (14%)	0.40

Data are presented as number (percentage), except for age, Acute Physiology and Chronic Health Evaluation II (APACHE II) score, arterial partial pressure of oxygen/fraction of inspired oxygen (PaO_2_/FiO_2_) ratio, Sequential Organ Failure Assessment (SOFA) score, hospital length of stay, and intensive care unit length of stay, which are presented as mean (standard deviation). ^a^Patients with pneumonia with septic shock were divided according to survival status at day 28; ^b^*P *value represents differences between survivors and non-survivors of pneumonia-related septic shock; ^c^organ dysfunction was determined on the day of enrollment.

### Plasma biomarker levels in patients with pneumonia and septic shock

**Figure 2:**
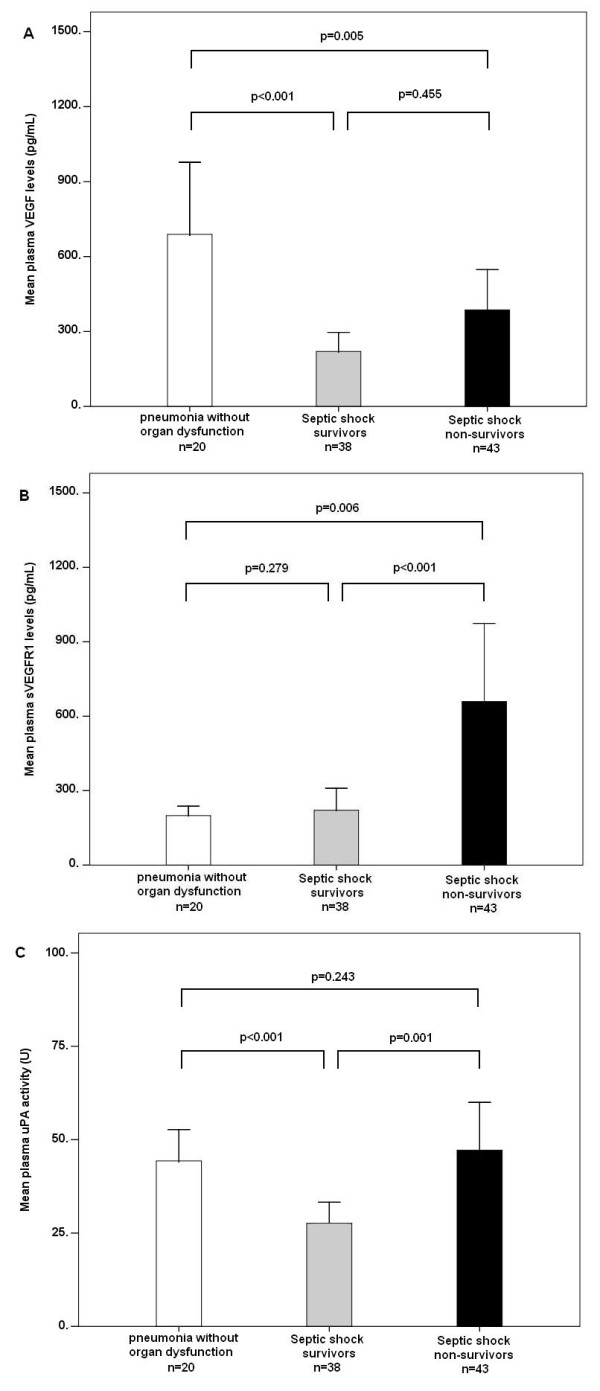
**Mean plasma biomarker levels in patient subgroups**. **(a) **Vascular endothelial growth factor (VEGF) and **(b) **soluble vascular endothelial growth factor receptor 1 (sVEGFR1) levels and **(c) **urokinase plasminogen activator (uPA) activity in patients with pneumonia without organ dysfunction (open bar), pneumonia-related septic shock survivors at day 28 (shaded bar), and non-survivors at day 28 (solid bar). Means and standard errors of the mean of plasma biomarker levels are shown. Significance was tested with the two-sided Mann-Whitney *U *test.

The mean VEGF level of the septic shock non-survivors was 386.5 ± 524.1 pg/mL, which was significantly lower than that of the patients with pneumonia without organ dysfunction (688.9 ± 616.9 pg/mL, *P *= 0.005) but comparable to that of the septic shock survivors (219.9 ± 232.1 pg/mL, *P *= 0.455) (Figure 2). The mean sVEGFR1 level of the septic shock non-survivors was 659.3 ± 1,022.8 pg/mL, which was significantly higher than that of the septic shock survivors (221.1 ± 268.9 pg/mL, *P *< 0.001) and those with pneumonia without organ dysfunction (199.3 ± 81.6 pg/mL, *P = *0.006). The mean plasma uPA activity of the septic shock non-survivors was 47.2 ± 40.6 units, which was significantly higher than that of the septic shock survivors (27.6 ± 17.2 units, *P *= 0.001) but comparable to that of the patients with pneumonia without organ dysfunction (44.3 ± 17.9 units, *P *= 0.243).

### Predictive value of plasma biomarkers for 28-day mortality

**Figure 3:**
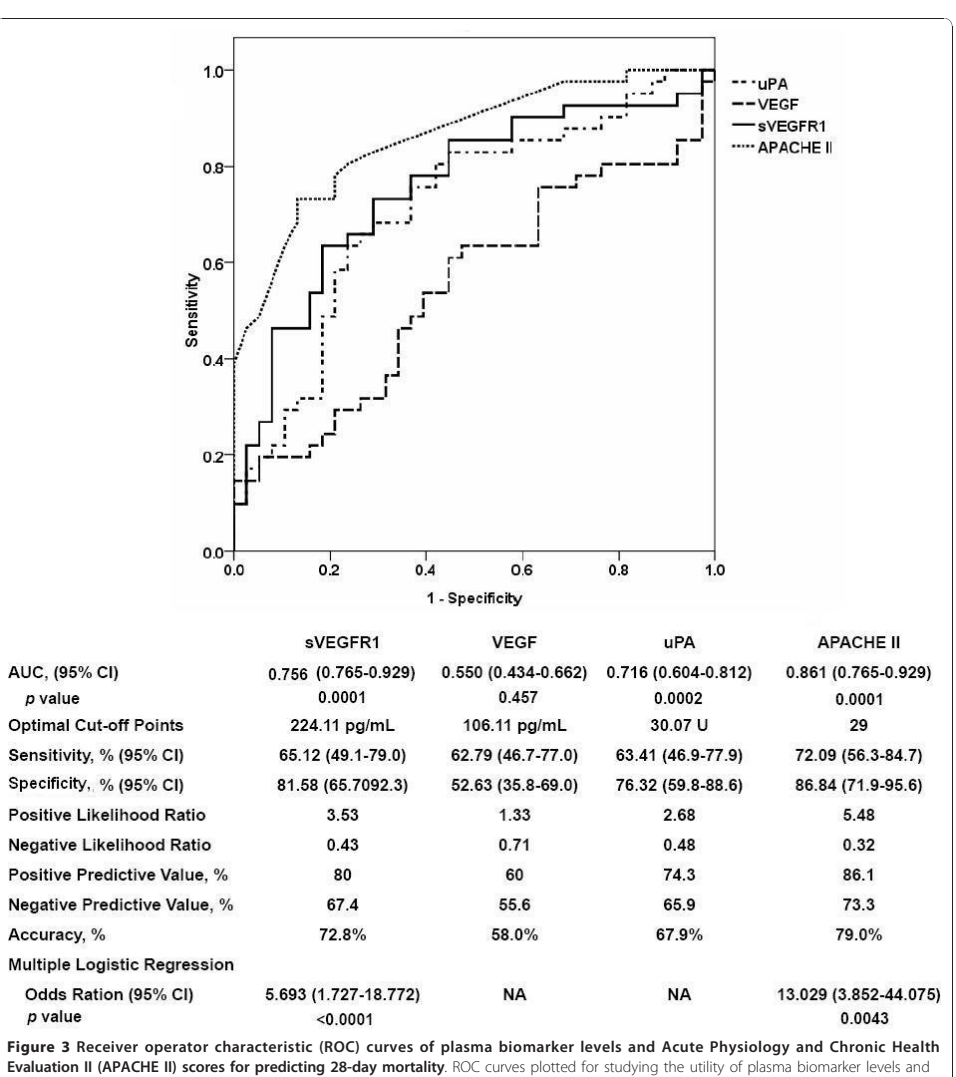
**Receiver operator characteristic (ROC) curves of plasma biomarker levels and Acute Physiology and Chronic Health Evaluation II (APACHE II) scores for predicting 28-day mortality**. ROC curves plotted for studying the utility of plasma biomarker levels and APACHE II scores in predicting 28-day mortality of pneumonia-related septic shock patients are shown. The areas under the ROC curves (AUCs) for soluble vascular endothelial growth factor receptor 1 (sVEGFR1), urokinase plasminogen activator (uPA), and APACHE II scores were significantly greater than 0.5. The optimal cutoff points for each biomarker and APACHE II score, along with their predictive values for the 28-day mortality of patients with pneumonia-related septic shock, are listed in the attached table. CI, confidence interval; VEGF, vascular endothelial growth factor.

The ROC curves of day-1 plasma levels of VEGF and sVEGFR1, uPA activities, and APACHE II scores in predicting 28-day mortality of septic shock patients are shown in Figure 3. APACHE II score, sVEGFR1 level, and uPA activity were good predictors of 28-day mortality, and the AUCs were 0.861 (95% confidence interval [CI] 0.765 to 0.929, *P *< 0.001), 0.756 (95% CI 0.647 to 0.846, *P *< 0.001), and 0.716 (95% CI 0.646 to 0.812, *P *< 0.001), respectively. The AUC of plasma VEGF level for 28-day mortality was 0.55 (95% CI 0.434 to 0.662, *P *= 0.46).

**Figure 4:**
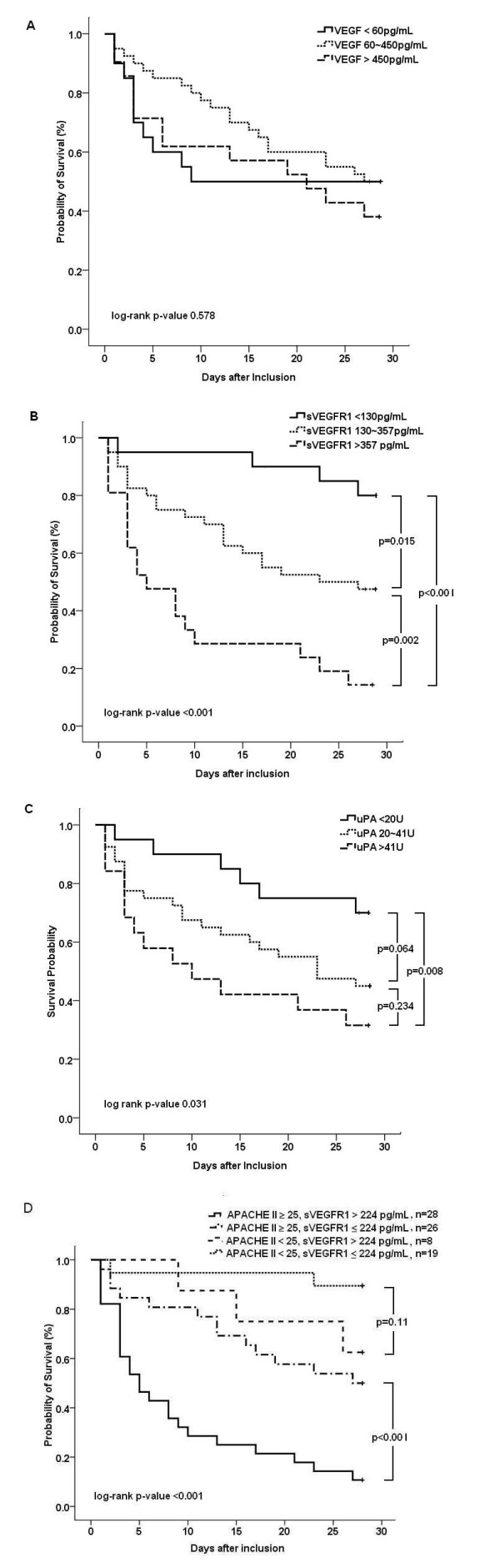
**Kaplan-Meier survival curves of patients with pneumonia-related septic shock, stratified by day-1 plasma biomarker levels**. Patients were categorized into three groups based on quartile levels of **(a) **vascular endothelial growth factor (VEGF), **(b) **soluble vascular endothelial growth factor receptor 1 (sVEGFR1), and **(c) **urokinase plasminogen activator (uPA), **(d) **combining sVEGFR1 levels and Acute Physiology and Chronic Health Evaluation II (APACHE II) scores. Significances were tested with the log-rank test.

The Kaplan-Meier analyses of survival according to quartile biomarker levels in septic shock patients are shown in Figure 4. Patients with higher plasma sVEGFR1 levels and uPA activities had significantly higher mortality (sVEGFR1, *P *< 0.001; uPA, *P *= 0.031) as compared with those with lower levels. The absolute difference in survival between the subgroups as represented by these biomarkers was evident from the early stage of septic shock. The survival curves overlapped between patients with different plasma VEGF levels (*P *= 0.58). Based on the optimal cutoff point of plasma sVEGFR1 level determined from the ROC curve (Figure 2), the Kaplan-Meier analysis of survival of the subgroups of patients divided by APACHE II scores and sVEGFR1 levels is shown in Figure 4d. In patients with an APACHE II score of at least 25, those with higher sVEGFR1 levels (>224 pg/mL) had a significantly higher mortality rate as compared with those with lower sVEGFR1 levels (*P *< 0.001). Similarly, in patients with an APACHE II score of less than 25, a trend toward higher mortality was found in those with higher sVEGFR1 levels (>224 pg/mL), but statistical significance was not achieved (*P *= 0.11).

Cox regression analysis models by quartile plasma biomarker levels, APACHE II scores, and comorbidities for the hazard ratio of mortality in septic shock patients are shown in Table 2. Both APACHE II score and increased plasma sVEGFR1 level were significant risk factors of 28-day mortality. The hazard ratios were 1.55 (*P *= 0.029) for every escalation of the sVEGFR1 quartile and 2.30 (*P *< 0.001) for that of APACHE II scores.

**Table 2:** Cox proportional hazards models for mortality prediction by comorbidities, biomarkers, and APACHE II score in patients with pneumonia-related septic shock

	Univariate Cox model		Multivariate Cox model	
		
Variables	HR (95% CI)	*P *value	HR (95% CI)	*P *value
Age	1.00 (0.97-1.03)	0.92		
Comorbidity				
Diabetes mellitus	1.15 (0.61-2.18)	0.66		
Obstructive airway disease	0.79 (0.38-1.63)	0.52		
Interstitial lung disease	0.72 (0.29-1.83)	0.50		
Chronic renal insufficiency	1.24 (0.60-2.58)	0.56		
Congestive heart failure	0.74 (0.26-2.05)	0.56		
sVEGFR1, quartile	1.97 (1.46-2.65)	<0.001	1.55 (1.05-2.30)	0.029
uPA, quartile	1.58 (1.19-2.10)	0.002	1.18 (0.77-1.79)	0.45
VEGF, quartile	1.09 (0.83-1.44)	0.52		
APACHE II, quartile	2.44 (1.78-3.35)	<0.001	2.30 (1.53-3.49)	<0.001

Relative risk and 95% confidence interval (CI) were derived from the Cox proportional hazards regression model. APACHE II, Acute Physiology and Chronic Health Evaluation II; HR, hazard ratio; sVEGFR1, soluble vascular endothelial growth factor receptor 1; uPA, urokinase plasminogen activator; VEGF, vascular endothelial growth factor.

### sVEGFR1 and uPA levels predict multi-organ dysfunction

**Figure 5:**
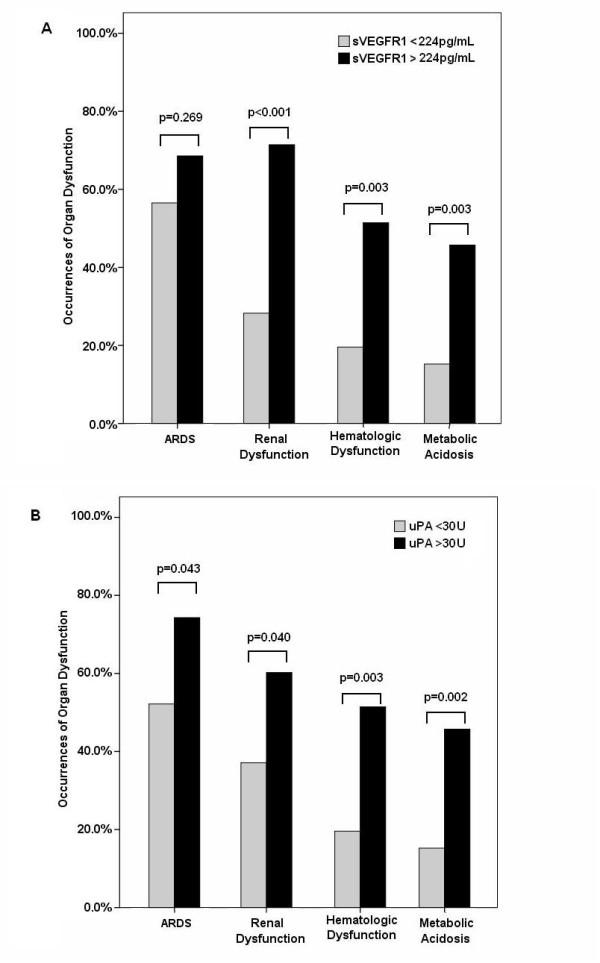
**Organ dysfunctions in patients with pneumonia-related septic shock**. The occurrence of organ dysfunction at the onset of septic shock was compared based on plasma **(a) **soluble vascular endothelial growth factor receptor 1 (sVEGFR1) levels and **(b) **urokinase plasminogen activator (uPA) activities above the optimal cutoff points (solid bars) and below the optimal cutoff points (shaded bars). Significance was tested by Pearson's chi-square test. ARDS, acute respiratory distress syndrome.

**Figure 6:**
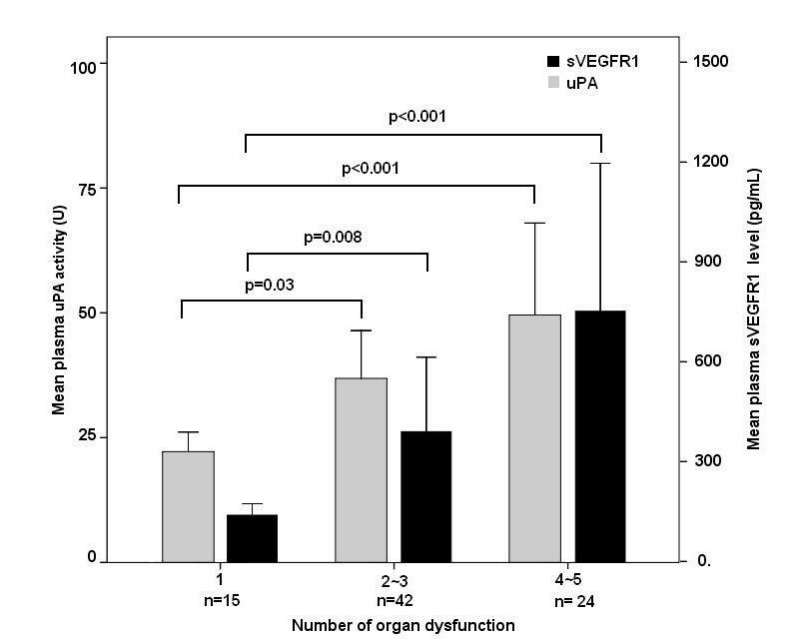
**Plasma soluble vascular endothelial growth factor receptor 1 (sVEGFR1) levels and urokinase plasminogen activator (uPA) activities in pneumonia-related septic shock patients with various numbers of organ dysfunctions**. Means and standard errors of the mean of the sVEGFR1 levels (solid bars) and uPA activities (shaded bars) are shown. Significance was tested with the two-sided Mann-Whitney *U *test.

The occurrence of organ dysfunction, including acute respiratory distress syndrome, renal dysfunction, metabolic acidosis, and hematologic dysfunction, based on biomarker levels is shown in Figure 5. When the day-1 plasma biomarker levels were stratified according to the optimal cutoff point determined from the ROC curve (Figure 3), patients with higher sVEGFR1 levels and uPA activities had significantly higher incidences of organ dysfunction. The mean plasma sVEGFR1 levels and uPA activities of patients with various numbers of organ dysfunctions are also shown in Figure 6. Patients with more organ dysfunctions had significantly higher plasma sVEGFR1 levels and uPA activities compared with those with only one organ dysfunction.

Multivariate analysis by quartile plasma biomarker levels, APACHE II scores, and comorbidities for the hazard ratio of multi-organ dysfunction are shown in Table 3. Increased plasma sVEGFR1 levels and uPA activities were independent risk factors for multi-organ dysfunction. The hazard ratios were 2.82 (*P *= 0.021) for every escalation of the sVEGFR1 quartile and 2.75 (*P *= 0.037) for that of the uPA quartile.

**Table 3:** Univariate and multivariate analyses of predictive factors for the presence of concomitant multi-organ dysfunction

	Univariate analysis		Multivariate analysis	
		
Variables	HR (95% CI)	*P *value	HR (95% CI)	*P *value
Age	1.01 (0.961.06)	0.76		
Comorbidity				
Congestive heart failure	0.19 (0.05-0.81)	0.016	0.13 (0.02-1.02)	0.052
Obstructive airway disease	0.41 (0.13-1.27)	0.12		
Chronic renal insufficiency	2.04 (0.41-10.05)	0.37		
Diabetes mellitus	1.24 ( 0.39-3.93)	0.72		
Interstitial lung disease	0.79 (0.19-3.33)	0.75		
sVEGFR1, quartile	2.71 (1.49-4.95)	<0.001	2.82 (1.17-6.81)	0.021
uPA, quartile	3.21 (1.64-6.32)	<0.001	2.75 (1.06-7.13)	0.037
VEGF, quartile	1.17 (0.70-1.95)	0.56		
APACHE II, quartile	1.74 (1.08-2.80)	0.017	1.09 (0.52-2.27)	0.82

Patients with pneumonia-related septic shock were enrolled for analysis. APACHE II, Acute Physiology and Chronic Health Evaluation II; CI, confidence interval; HR, hazard ratio; sVEGFR1, soluble vascular endothelial growth factor receptor 1; uPA, urokinase plasminogen activator; VEGF, vascular endothelial growth factor.

## Discussion

This study demonstrated that plasma sVEGFR1 level and uPA activity are valuable predictors of 28-day mortality in patients with pneumonia-related septic shock. The role of the initial plasma VEGF level in predicting 28-day mortality and organ dysfunction was insignificant. In multivariate Cox regression analysis, the plasma sVEGFR1 level was the most significant plasma biomarker for 28-day mortality. We also found sVEGFR1 level and uPA activity to be the independent predictors of multi-organ dysfunction. The role of these biomarkers in the pathogenesis of sepsis deserves further exploration.

VEGF has been reported to be an important mediator in increasing vascular permeability and exacerbating hemodynamic instability [9]. Signaling of the VEGF pathway also contributes to the proliferation and survival of endothelial cells [10]. Previous studies have concluded that plasma VEGF levels are elevated in patients with sepsis (as compared with healthy controls) and have good correlation with organ dysfunction and disease severity [14-16,25]. However, studies evaluating the predictive value of plasma VEGF levels with respect to mortality have yielded controversial results [15,25]. In our study, there was no statistical difference in plasma VEGF levels between the survivors and non-survivors of pneumonia-related septic shock. The results of our study, and those of previous reports, indicate that the complex effect of VEGF in sepsis remains to be clarified.

Our study showed that the plasma sVEGFR1 level, unlike VEGR level, was an independent predictive factor for mortality and the presence of concomitant multi-organ dysfunction. More importantly, we demonstrated that, as compared with patients with high APACHE II scores (≥25) and lower sVEGFR1 levels (≤224 pg/mL), those with high APACHE II scores (≥25) and higher sVEGFR1 levels (>224 pg/mL) had significantly higher mortality. Probably owing to relatively small sample size, there was no statistical difference in mortality rate between patients with higher or lower sVEGFR1 levels in the subgroup of patients with lower APACHE II scores (<25); nevertheless, a trend toward higher mortality in those with higher sVEGFR1 levels existed. Our findings indicated that combining the APACHE II score and sVEGFR1 level yield better discriminative power in mortality prediction. This further clarifies the clinical application of plasma sVEGFR1 level in patients with septic shock.

The soluble form of VEGFR1 is a splicing variant of VEGFR1 and exerts a negative influence on VEGF signaling. The role of sVEGFR1 in sepsis is probably dual, involving endothelial cell damage and an anti-inflammatory effect. The upregulation of VEGF and VEGFR1 on the cell surface is widely reported after endothelial injury and functions as a protective mechanism that enhances vascular remodeling and promotes re-endothelialization [26,27]. The inhibitory effect of sVEGFR1 in VEGF signaling may hamper the process of endothelial repair. Therefore, the elevation of plasma sVEGFR1 level in sepsis may lead to disease progression and organ dysfunction. Meanwhile, VEGF has been reported to have pro-inflammatory properties; an *in vitro *study showed VEGFR1 to be involved in the migration of monocytes/macrophages [28]. As a negative counterpart in VEGF signaling, elevation of sVEGFR1 will lead to an anti-inflammatory state; however, uncontrolled elevation of sVEGFR1 may also cause profound immune depression and lead to a worse outcome in patients with septic shock. Therefore, the opposing actions of sVEGFR1 on VEGF may have contributed to the inconsistent results regarding the role of VEGF in sepsis in the present and previous studies.

The role of uPA in the pathogenesis of sepsis is probably complex. Through the conversion of plasminogen to plasmin, uPA may play an antithrombotic role in sepsis. uPA also has pro-inflammatory effects, including cytokine induction, neutrophil activation, and enhancement of lipopolysaccharide-related inflammation [19,29]. The interaction between uPA and VEGF has been described; pro-uPA can be activated by VEGF on endothelial cells and is involved in the VEGF-related angiogenic process [30]. Behzadian and colleagues [21] found that the delayed phase of VEGF-induced permeability change could be blocked by anti-uPA antibodies *in vitro *and concluded that uPA is an important mediator in the process of VEGF-mediated permeability change. The role of uPA, despite its diverse effects, in patients with septic shock remains to be determined. A few previous studies have reported increased uPA activity in patients with severe infection or sepsis [31,32]. In this study, for the first time, we identified uPA activity as an independent predictor of concomitant multi-organ dysfunction in patients with septic shock. In addition to antithrombotic effects, the elevation of uPA in sepsis may involve the VEGF pathway, which includes endothelial cell repair and migration, angiogenesis, and vascular permeability change.

The major limitations of this study include the small number of cases and low patient population diversity. A relatively small number of cases may lead to a wide range of standard deviations of biomarker levels. Only patients with pneumonia-related septic shock were enrolled, and most of them were older males with profound disease severity. The homogeneity of the patients reflects the characteristics of our hospital (that is, a veterans hospital). Nevertheless, the low diversity minimizes the confounding factors in the patient population and also allows our findings to be reliably applied to older pneumonia patients with critical illnesses. Another limitation is that blood samples were collected only from the first day of shock onset; therefore, the variation trend of these biomarkers in the course of septic shock could not be evaluated in the present study.

## Conclusions

Our results suggest that sVEGFR1 level determined in the early phase of pneumonia-related septic shock is a promising predictor for multi-organ dysfunction and 28-day mortality. The immunosuppressive effect of sVEGFR1 makes it a novel marker of immunoparalysis in the pathogenesis of sepsis. However, the correlation between sVEGFR1 and uPA and their roles in endothelial dysfunction deserve more investigation. Further studies with a larger number of patients and diverse infectious origins are warranted to identify the role of these biomarkers in septic shock.

## Key messages

• Plasma soluble vascular endothelial growth factor 1 (sVEGFR1) level in the early phase of pneumonia-related septic shock is an independent predictor of 28-day mortality and the presence of concomitant multi-organ dysfunction.

• Combining Acute Physiology and Chronic Health Evaluation II (APACHE II) score and plasma sVEGFR1 level can help in identifying patients with an extremely high risk of mortality.

• Plasma urokinase plasminogen activator activity in the early phase of pneumonia-related septic shock is an independent predictor of the presence of concomitant multi-organ dysfunction.

• The predictive value of plasma vascular endothelial growth factor level for 28-day mortality and concomitant multi-organ dysfunction is limited in the present study.

## Abbreviations

APACHE II: Acute Physiology and Chronic Health Evaluation II; AUC: area under the curve; CI: confidence interval; ICU: intensive care unit; ROC: receiver operating characteristic; SOFA: Sequential Organ Failure Assessment; sVEGFR1: soluble vascular endothelial growth factor 1; uPA: urokinase plasminogen activator; VEGF: vascular endothelial growth factor; VEGFR1: vascular endothelial growth factor receptor 1.

## Competing interests

The authors declare that they have no competing interests.

## Authors' contributions

K-YY and J-YF helped to design the study, write the manuscript, enroll patients, and collect samples. Y-CL, C-SC, and R-PP helped to design the study and write the manuscript. K-TL helped to enroll patients and collect samples. Y-CC was responsible for statistical analysis. All authors read and approved the final manuscript.

## Supplementary Material

Additional file 1 **Criteria for organ dysfunction**. ARDS, acute respiratory distress syndrome. Click here for file
