# Supplementary material for: Plasma soluble vascular endothelial growth factor receptor-1 levels predict outcomes of pneumonia-related septic shock patients: a prospective observational study
Source: Crit Care. 2011 Jan 10;15(1):R11. doi: 10.1186/cc9412 (PMC3222041; doi:10.1186/cc9412)
Supplement: Additional file 1 — Criteria for organ dysfunction. ARDS, acute respiratory distress syndrome. [file cc9412-S1.DOC]

Appendix 1.

Criteria for organ dysfunction

| Organ dysfunction | Criteria |
| --- | --- |
| Kidney dysfunction | Urine output <0.5 ml/kg of body weight/hr for 1 hour, despite adequate fluid resuscitation |
| Hematologic dysfunction | Platelet count < 80,000/mm^3^, or having decreased 50% in the 3 days preceding enrollment |
| Metabolic acidosis | pH ≤ 7.30 or the base deficit ≥ 5.0 mmol/liter in association with a plasma lactate level > 1.5 times the upper limit of the normal value |
| ARDS | Acute onset  Bilateral infiltrates on chest radiograph  Pulmonary artery wedge pressure < 18 mmHg or lack of clinical evidence of left ventricular failure  PaO_2_/FiO_2_ < 200mmHg |

ARDS, acute respiratory distress syndrome
